# Enhancing battery thermal management system using the emulsion of encapsulated phase change material and water in a mini helical tube

**DOI:** 10.1038/s41598-025-30112-3

**Published:** 2025-12-03

**Authors:** Shimaa A. Hussien, Ali B. M. Ali, Omar J. Alkhatib, Ahmad Hamedian, Ibrahim Mahariq

**Affiliations:** 1 Electrical Department, Faculty of Engineering, Princess Nourah Bint Abdulrahman University, P.O. Box 84428, 11671 Riyadh, Saudi Arabia; 2 Advanced Technical College, University of Warith Al-Anbiyaa, Karbala, Iraq; 3 Architectural Engineering Department, College of Engineering, UAE University, Al Ain, United Arab Emirates; 4 Department of Mechanical Engineering, Duy Tan University, Da Nang, Vietnam; 5 Department of Mathematics, Saveetha School of Engineering, SIMATS, Saveetha University, Chennai, Tamil Nadu 602105 India; 6 College of Engineering and Architecture, Gulf University for Science and Technology, Mishref, Kuwait; 7 University College, Korea University, Seoul, 02481 South Korea; 8 Department of Medical Research, China Medical University Hospital, China Medical University, Taichung, Taiwan; 9 Present Address: Najjad Zeenni Faculty of Engineering, Al-Quds University, Jerusalem, Palestine

**Keywords:** Battery thermal management system, Encapsulated phase change material, Emulsion, Mini helical tube, CFD simulation, Energy science and technology, Engineering, Materials science

## Abstract

A key challenge in limiting the performance and safety of lithium-ion batteries (LIB), particularly at high discharge rates, is excessive heat generated during operation. The purpose of this study is to investigate the innovative use of encapsulated phase change materials (EPCMs) in a cooling system of a mini helical tube (MHT) in order to improve battery thermal management (BTM) in lithium-ion batteries. The results reveal that increasing the Reynolds number (Re) from 50 to 100 at 4% EPCM concentration (C) results in a reduction of 5.26 K and 2.48 K at the average outlet temperature (To) and the LIB temperature (TLIB). Furthermore, raising the C from 0 to 4% resulted in a 1.39 K decrease in TLIB at Re = 100. Moreover, the Nusselt number (Nu) is significantly improved with both Re and C, with Nu increasing by 36.35% when Re is changed from 50 to 100, while Nu increases by 178.4% when EPCM is added at Re = 50.

## Introduction

A Battery Thermal Management System (BTMS) regulates the temperature of the battery cells during operation and charging in order to ensure safety, performance, and longevity^1^. As overheating can trigger thermal runaway, sustained deviations from the optimal range reduce efficiency and accelerate degradation^2,3^. The use of an effective BTMS minimizes internal resistance losses and extends the lifespan of LIBs^4^. BTMS strategies include passive and active approaches^5^. The passive BTMS principles (PBTMS) involve managing heat without the use of external energy, such as the use of heat sinks^6^, heat pipes^7^, configuration optimization^8^, and PCMs^9^ that absorb heat during melting.

Several studies have investigated heat pipes (HPs) for cooling cylindrical Li-ion cells by transferring heat from the circumferential surface to a remote sink without active pumps^10,11^. Using a novel HP–cell contactor design, peak temperature can be reduced to 42.2 degrees Celsius at 3 °C discharge, thus improving heat extraction efficiency by 63.5%^10^. The use of mini-channel heat sinks is an effective approach for active BTMS^12^. The mini-channel heat sinks provide high surface area-to-volume ratios in a compact footprint^13^, which ensures efficient convection heat removal while ensuring uniform temperature distribution across cells^14^. Due to their small size, they require less coolant and pumping power^15^, making them an attractive option for applications that are space- and weight-sensitive, such as electric vehicles^16^. Mini-channel cooling systems are easily scalable, can be adapted to various cell geometries, and can be integrated into hybrid cooling systems^17^.

PCMs can store and release large amounts of latent heat during solid-liquid transitions^18^, facilitating effective thermal energy storage and temperature regulation^19^. Typically, they can be classified as organic (i.e., paraffins, fatty acids)^20^, inorganic (i.e., hydrated salts)^21^, or eutectic mixtures^22^, which each offers distinct TPPs and performance-cost trade-offs. The encapsulation process has advanced PCMs by enclosing them within protective shells made of polymers^23^, metals^24^, and ceramics^25^, resulting in encapsulated PCMs (EPCMs). This approach prevents leakage, enhances thermal stability^26^, and ensures performance is maintained over repeated phase change cycles^27^. The EPCMs can be integrated into compact or dynamic systems without compromising structural or thermal integrity^28^, making them suitable for applications such as electronics cooling^29^, energy storage^30^, and temperature-sensitive product transportation^31^. In heat transfer applications, EPCMs act as thermal buffers^32^, absorbing excess heat during high-load conditions and dissipating it when temperatures drop^33^. Dispersing EPCMs in a base fluid to form an emulsion further enhances convective heat transfer by combining sensible and latent heat transfers^34^. Earlier studies have demonstrated significant improvements in heat transfer and performance index using EPCM-based emulsions in forced convection systems^35–37^. The study^38^ synthesizes EPCM nanoparticles with a diameter of 250–350 nm and eicosane cores. When used in a pipe, NEPCM-water produced significant improvements in heat transfer and performance index by 70% and 45%, respectively, particularly at low Re, despite viscosity impacts at higher flow rates. Additionally, the study^39^ introduced convective drying of moist porous materials using micro EPCM. In numerical investigations, PCM significantly enhanced drying efficiency, resulting in a 70.2% increase in heat transfer with a 50% PCM volume fraction. Recent studies have highlighted advances in the design of BTMSs. An aluminum mini-channel system was found to maintain LIB temperatures below 27.8 °C at 1 C while using a minimal amount of pumping power^40^. A hybrid PCM-liquid cooling method for prismatic packs reduced peak temperature by 16.2 K at 3 C and reduced power consumption by 68%^41^. In cylindrical LIBs, integrating liquid cooling with air cooling reduces the maximum temperature to 304.98 K and the temperature difference to 4.13 K, with additional gains from increased airflow^42^. Based on these findings, it is evident that multi-mode cooling strategies are a viable option for BTMS with high performance.

Several studies have demonstrated that mini-channels can regulate LIB temperatures effectively due to their high surface-to-volume ratio^40^, while EPCM–water emulsions can significantly increase heat transfer in thermal systems due to their high latent heat capacity^38^. However, there has been no investigation of their combined application in BTMS. The present study aims to fill this gap by utilizing CFD to investigate a novel configuration: an MHT wrapped directly around a cylindrical LIB and cooled with EPCM-water emulsion. The design maximizes thermal contact and leverages latent heat storage to deliver a compact, high-performance BTMS solution.

## Problem and schematic

**Fig. 1:**
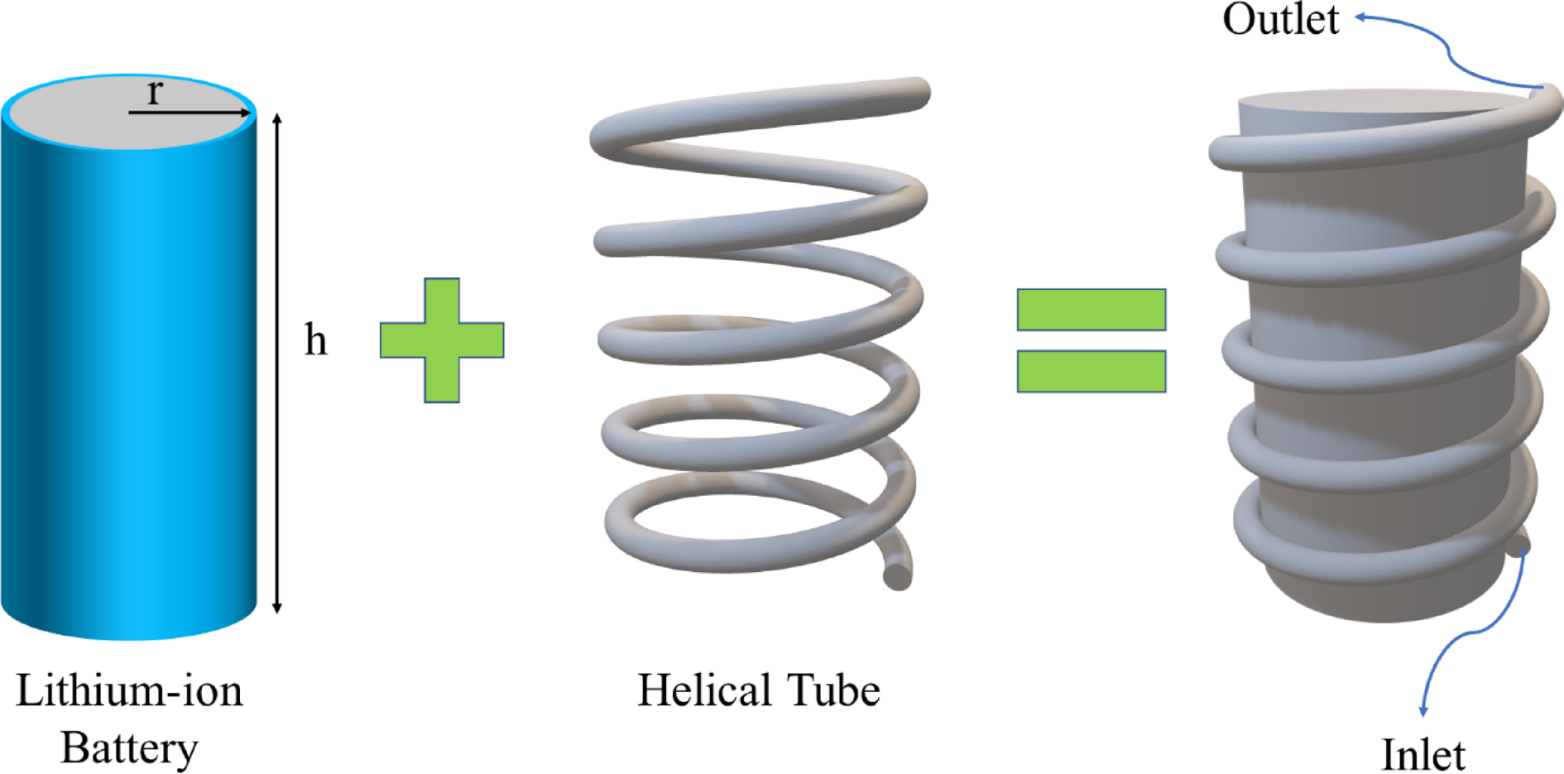
shows a schematic of the proposed BTMS configuration, which features a cylindrical LIB encircling an MHT.

Cooling batteries effectively at high C-rates continues to pose a major challenge, as excessive heat generation compromises safety, performance, and lifespan. Miniature cooling devices offer efficient thermal regulation due to their high surface-to-volume ratio. The objective of this numerical study is to propose a compact MHT that is wrapped directly around an 18,650 cylindrical LIB in order to maximize thermal contact and heat removal (Fig. 1). A summary of the key specifications of the 18,650 cells can be found in Table 1. This type of LIB has a safe operating temperature range of 293 K to 313 K, as specified in Table 1.

**Table 1:** Specifications of the 18,650 cylindrical libs used in this study.

Parameter	Value	Unit
Cathode	LiMn_4_O_4_	–
Anode	Carbon	–
Diameter	18	mm
Length	65	mm
Density	2007.7	$\:\frac{\text{kg}}{{\text{m}}^{3}}$
Specific heat capacity	837.4	$\frac{J} {\text{kg K}}$
Nominal capacity	3.6	$\text{Ah}$
Nominal current	3.6	$\text{A}$
Internal resistance	0.1	$\:{\Omega\:}$
Safe temperature	293–313	K

In this study, the 18,650 LIB operates at a discharge rate of three C, which produces a significant amount of heat. The proposed MHT consists of five coils around the LIB, with *r* = 2 mm, H = 65 mm, and *P* = 13 mm, in order to maximize the contact area between the coils and the LIB. MHTs are assumed to be securely bonded to LIBs using high-conductivity thermal pastes (like silicone-based ones) to minimize interfacial resistance. An EPCM-water emulsion served as a cooling medium, entering at the base and exiting at the top of the LIB (Fig. 1). As the emulsion flows, EPCM cores begin to melt at their phase-change temperature, absorbing latent heat and increasing the efficiency of the coolant. The use of this mechanism results in a significant improvement in heat removal efficiency compared to the use of pure water cooling in this study.

## Formulation

As part of the present CFD simulation, formulations are developed for various components, including the PDEs and the relationships governing the water-EPCM emulsion. The flow is assumed to be incompressible since the liquid emulsion experiences minimal pressure variations, resulting in negligible compressibility within the MHT. The analysis focuses on steady-state conditions, which is justified by the small system size, which enables rapid stabilization of the system. The flow of the emulsion is considered laminar due to the small diameter and low velocity of the MHT. The EPCMs are assumed to be dispersed uniformly in the water, maintaining homogeneity throughout the flow of the liquid. External forces, such as magnetic fields from the LIB and gravitational effects, are not taken into account. Based on these assumptions, the continuity, momentum, and energy equations are formulated as follows:^34^:

1 $$\:\nabla\:\cdot \varvec{V}=0$$

2 $$\:{\rho\:}_{e}\left(\varvec{V} \cdot \nabla\:\right)\varvec{V}=-\nabla\:p+{\mu\:}_{e}\nabla\:\cdot\left(\nabla\:\varvec{V}\right)$$

3 $$\:\nabla\:\cdot\left({\rho\:}_{e}{C}_{p,\:e}T\varvec{V}\right)=\nabla\:\cdot \left({k}_{e}\nabla\:T\right)$$

The left-hand side of the governing momentum equation represents convection, whereas the right-hand side represents the combined effects of pressure gradients and viscous stresses. Similarly, the energy equation describes the balance between convective energy transfer and conduction heat transfer within emulsion flows. To determine the effective TPPs of a water-EPCM emulsion, emulsion rules are applied. The density and specific heat capacity of water-EPCM emulsion are calculated by averaging the volume fractions of the base fluid (water) and EPCM particles^38^. The Hamilton–Crosser model^37^ is commonly used to estimate the effective thermal conductivity of heterogeneous emulsions with spherical or near-spherical inclusions. The Brinkman model^36^ is employed to determine the viscosity of the suspension, providing a reliable correction for diluted particle concentrations. The correlations presented here are frequently used in studies of nanoparticle- and PCM-based emulsions^36^, and their use here ensures consistency with previous validated methods.

4 $$\:{\rho\:}_{e}=\left(1-C\right){\rho\:}_{bf}+C{\rho\:}_{EPCM}$$

5 $$\:{k}_{e}=\frac{{k}_{EPCM}+2{k}_{bf}+2C({k}_{EPCM}-{k}_{bf})}{{k}_{EPCM}+2{k}_{bf}-C({k}_{EPCM}-{k}_{bf})}{k}_{bf}$$

6 $$\:{\mu\:}_{e}=\frac{{\mu\:}_{bf}}{{\left(1-C\right)}^{2.5}}$$

7 $$\:{\left(\rho\:{C}_{p}\right)}_{e}=\left(1-C\right){\rho\:}_{bf}{C}_{p,\:bf}+C{\rho\:}_{EPCM}{C}_{p,\:EPCM}$$

For evaluation of the equivalent TPPs of the EPCM, both the solid shell and the PCM core must be taken into account. The EPCM is modeled as a composite particle, in which the shell provides structural stability while the core provides latent heat storage. The effective density and specific heat capacity of the EPCM are calculated using linear mixing rules that are based on the weight (or volume) fractions of the shell and PCM core^36^. Effective thermal conductivity is evaluated using a parallel thermal resistance model assuming spherical capsule geometry, which is a widely used approximation in the study of heat transfer in encapsulated PCM systems. As a result, the EPCM density, specific heat capacity, and thermal conductivity can be expressed as follows^34^:

8 $$\:{\rho\:}_{EPCM}=\frac{(1+\omega\:){\rho\:}_{c}{\rho\:}_{s}}{{\rho\:}_{s}+\omega\:{\rho\:}_{c}}$$

9 $$\:{k}_{EPCM}=\frac{{d}_{s}}{\frac{{d}_{c}}{{k}_{c}}+\frac{{d}_{s}-{d}_{c}}{{k}_{s}}},\:{d}_{c}={d}_{s}{\left(\frac{{\rho\:}_{s}}{{\rho\:}_{s}+\omega\:{\rho\:}_{c}}\right)}^ {1 \mathord{\left/ {\vphantom {1 3}} \right. \kern-\nulldelimiterspace} 3}$$

10 $$\:{C}_{p,\:EPCM}=\frac{({C}_{p,\:c}+\omega\:{C}_{s}){\rho\:}_{c}{\rho\:}_{s}}{({\rho\:}_{s}+\omega\:{\rho\:}_{c}){\rho\:}_{EPCM}}$$

Finally, it is necessary to derive a formula for the PCM core’s effective heat capacity. This calculation must capture both sensible heat (resulting from the PCM’s specific heat capacity) and latent heat released during the solid–liquid phase transition. To account for melting gradually over a finite temperature range, latent heat is distributed over the phase-change temperature range. Several approaches are available, such as enthalpy-based methods, piecewise linear functions, and smooth transition functions^31^. This study employs a sine-based smoothing function to ensure numerical stability and to eliminate discontinuities in the heat capacity profile across the melting zone^36^. Accordingly, the equivalent heat capacity of the PCM core is defined as follows^34^:

11 $$C_{{p,~c}} = C_{{p,cl}} + \left\{ {\frac{\pi }{2}\left( {\frac{h}{{T_{{mr}} }} - C_{{p,cl}} } \right)\left( {\sin \pi \frac{{T - \left( {T_{m} - {{T_{{mr}} } \mathord{\left/ {\vphantom {{T_{{mr}} } 2}} \right. \kern-\nulldelimiterspace} 2}} \right)}}{{T_{{mr}} }}} \right)} \right\} \times \left\{ {\begin{array}{*{20}l} 0 \hfill & {if\;T < T_{m} - \frac{{T_{{mr}} }}{2}} \hfill \\ 1 \hfill & {if~\;T_{m} - \frac{{T_{{mr}} }}{2} < T < T_{m} + \frac{{T_{{mr}} }}{2}} \hfill \\ 0 \hfill & {if\;T > T_{m} + \frac{{T_{{mr}} }}{2}} \hfill \\ \end{array} } \right.$$

As part of the evaluation of the equivalents for both the emulsion and EPCM, the intrinsic TPPs of the individual components are required, namely the base fluid (water), the PCM core (n-octadecane), and the encapsulating shell material (PMMA). This includes density, thermal conductivity, dynamic viscosity, specific heat capacity, latent heat, and melting characteristics. A summary of the adopted values, based on rigorous experimental and numerical studies^36^, is presented in Table 2, with each TPP expressed in consistent SI units to facilitate understanding.

**Table 2:** The TPPs of water, n-octadecane, and PMMA^36^.

Parameter	Water	*n*-$\:\:\varvec{o}\varvec{c}\varvec{t}\varvec{a}\varvec{d}\varvec{e}\varvec{c}\varvec{a}\varvec{n}\varvec{e}$	PMMA
$\:{\rho\:}$	996.5	841	1185
$\:{k}$	0.63	0.15	0.2
${\mu\:}$	$\:850\times\:{10}^{-6}$	–	–
$\:{{C}}_{{p}}$	4181	2127	1466
${h}$	–	243.5	–
$\:{{T}}_{{m}}$	–	301	–
$\:{{T}}_{{m}{r}}$	–	1.5	–

The specification of boundary conditions is one of the most important steps in CFD simulations. In the present study, the emulsion is introduced into the MHT through the inlet at a uniform velocity determined by the Re. At the outlet, a fully developed (zero-gradient) condition is applied to the momentum equation. The MHT wall is subjected to a no-slip condition, ensuring that the first layer of the emulsion adheres to the surface. In the energy equation, the emulsion entered the MHT at a uniform temperature of 300 K and exited under a zero-gradient condition. Accordingly, the MHT wall is assigned a constant heat flux, which represents the heat generated by the LIB as a result of internal resistance and electrochemical reactions. It is calculated as follows:

12 $$\:Q=R{I}^{2}+I{T}_{ref}dET$$

where Q represents the heat generated by the LIB due to both internal resistance and electrochemical reactions, with C-rate constant at 3, and reference temperature 300 K. The electrochemical heat generation is expressed as dET, which is 0.4 mV/K for LIBs^38^. Assuming strong thermal contact between the LIB and MHT through thermal paste, the heat flux is transferred directly to the MHT wall rather than modeling full conjugate heat transfer. As a result, this approach simplifies the numerical model while retaining physical accuracy for evaluating the performance of the coolant side.

## Solving approach and validation

**Fig. 2:**
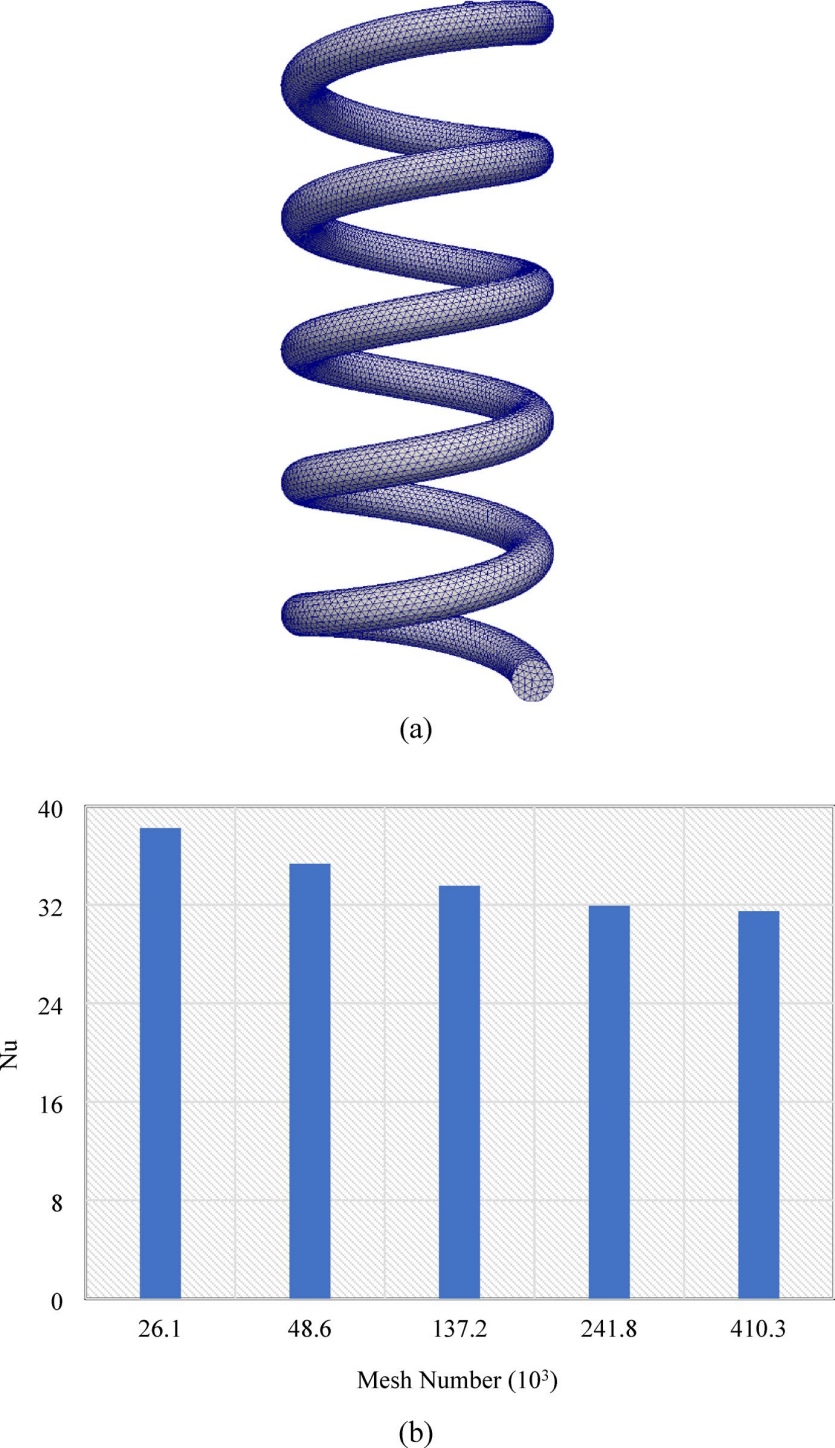
(**a**) The illustration of the tetrahedral coarse mesh used to discretize the MHT (**b**) and the plot of the Nu values for various mesh sizes to assess mesh independence.

A numerical approach is applied to investigate BTMS. The FVM within CFD is used to discretize both the governing equations and the simulation domain, enabling detailed analysis of heat transfer between the LIB and the MHT. Convective terms are discretized using a first-order upwind scheme, while conductive or diffusive terms are discretized using a second-order central scheme. A SIMPLE algorithm is used to couple velocity and pressure fields. Numerical convergence is ensured by establishing residual thresholds of 10⁻⁶ for the velocity and pressure equations, and 10⁻⁷ for the temperature equation. The computational domain is meshed using a tetrahedral grid (Fig. 2a). Mesh quality is verified through assessment of skewness (maintained below 0.3) and aspect ratio (constrained to values less than 5), whereas mesh independence is systematically evaluated by utilizing five discretization levels with element counts of 26,100, 48,600, 137,200, 241,800, and 410,300 at Ra = 100 and concentration (*C*) of 4%. The corresponding Nusselt number (*Nu*) values are presented in Fig. 2b. Analysis of these results reveals that the percentage discrepancies in *Nu* between successive mesh refinement levels (with element counts ranging from 26,100 to 410,300) decrease systematically, yielding values of 7.46%, 4.63%, 2.83%, and 0.44%, respectively. In other words, Nu exhibits convergent behavior with progressive mesh refinement, demonstrating variations of less than 3% between discretizations of 241,800 and 410,300 elements, confirming the independence of meshes. Consequently, 241,800 elements were selected as the optimal compromise between numerical accuracy and computational efficiency. This methodological decision is substantiated by rigorous quantitative analysis, which demonstrates that by increasing the mesh resolution from 241,800 to 410,300 elements, computational expenditure is enhanced by 76% while Nu values only increase by 0.44%. Such a diminutive enhancement in numerical accuracy does not justify the considerable additional computational resources that would be required for implementation. Moreover, this selection is supported by quantitative assessment, which indicates that increasing the mesh resolution from 241,800 to 410,300 elements results in a 76% increase in computational expenditure, resulting in only a marginal improvement in Nu values—a marginal increase, which does not justify the significant additional computational resources required.

The validity of the CFD model is confirmed by comparison with established numerical and experimental results. For this purpose, the results are compared with those of a study^36^, which investigated EPCM flow in a simple tube with a constant wall temperature. Several specific conditions are selected from that study, including a mass fraction of 0.02 and an X of 20 for a variety of Re values. Table 3 compares the present simulation with the reference study and indicates a maximum deviation of less than 3.25%. Consequently, this agreement confirms the accuracy and reliability of the numerical model for predicting convective heat transfer based on EPCMs.

**Table 3:** A comparison of the Nu for work^36^ and our simulation for different Re.

Re	Work^36^	This work
100	2.28	2.231
300	4.45	4.324
500	5.17	5.072
700	5.32	5.147

An additional validation step was performed to further confirm the accuracy of the simulations for BTMS applications. In this step, the results of this study have been compared with those reported in study^43^, which investigated different Reynolds numbers as a means of cooling a cylindrical LIB pack using a nanofluid. A comparison of the predicted Nusselt numbers is presented in Table 4. The differences between the two studies are small, with relative errors ranging from 2.05% to 2.74%, thus supporting the accuracy of the current numerical approach.

**Table 4:** Comparison of Nu obtained by the present simulations with those reported in the benchmark study^43^.

	Study^43^	Present work	Error (%)
Re = 1000	10.45	10.664	2.05
Re = 1500	11.73	11.758	2.41
Re = 2000	13.36	13.72	2.74

## Results and discussions

**Fig. 3:**
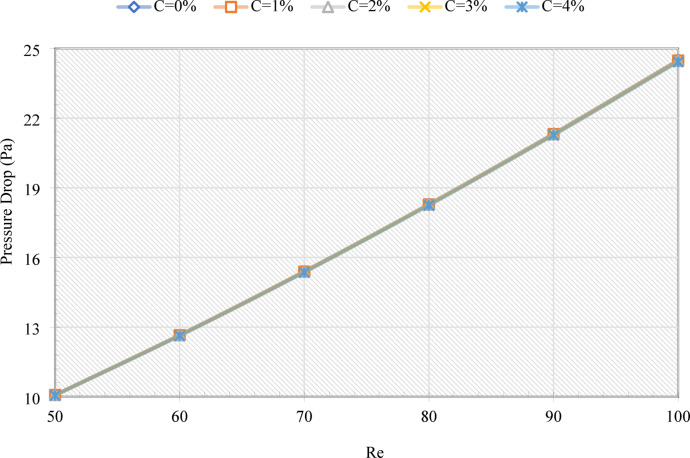
The plot of the pressure drop for various Re and C for the proposed cooling system for BTMS.

The BTMS is investigated using an MHT, through which an emulsion of water and EPCM flows to regulate the TLIB. There are several parameters that have a significant impact on the performance of a system, including Re and C. The value of Re determines the flow regime and the capacity of convection, with higher values generally resulting in better heat transfer. As C regulates the TPPs of the emulsion, optimizing them is essential for effective temperature control. It is also important to evaluate the pressure drop, as it represents flow resistance and pumping requirements, directly affecting the efficiency of the system. Among the additional indicators are T_LIB_ and To, which assess cooling effectiveness and aid in the prevention of overheating. The Nu is examined in order to quantify convective enhancement, while TLIB distributions are examined in order to identify hotspots and ensure uniform heat dissipation throughout the system. Finally, the melting behavior of EPCM cores is considered, since latent heat absorption during phase transition offers additional thermal buffering, thereby extending the cooling capacity of the system during varying operating conditions. Figure 3 illustrates the pressure drop within the MHT as a function of emulsion viscosity and MHT curvature. The pressure drop increases almost linearly with Re, as high fluid velocity increases shear stress and frictional losses. For example, increasing Re from 50 to 100 results in a 2.5-fold increase in pressure drop. The variation of C results in only slight changes in the viscosity of the emulsion throughout the studied range. While the pressure penalty increases with Re, the enhanced heat transfer (Nu) results in a net performance improvement. .

**Fig. 4:**
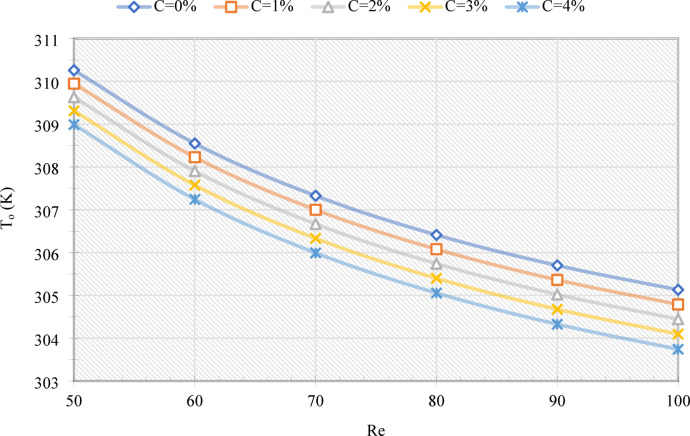
The T_o_ from the MHT for various Re and C values.

**Fig. 5:**
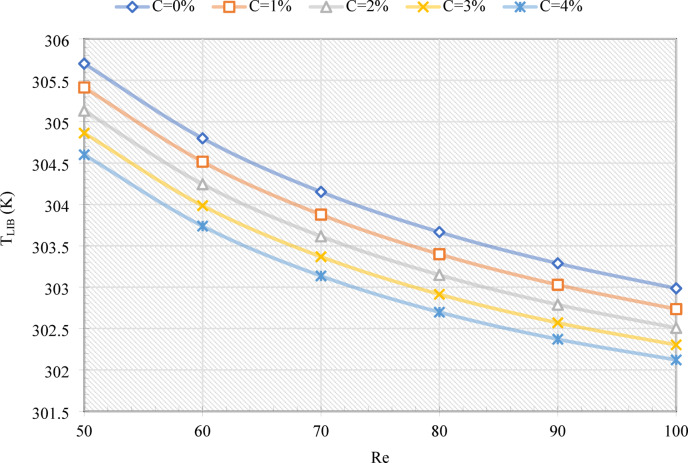
The T_LIB_ on the MHT for various Re and C values.

Figure 4 illustrates the T_o_ of the MHT as a function of the Re and C values. With increasing Re, T_o_ decreases consistently, confirming the enhancement of convective heat transfer. Increasing Re from 50 to 100, for example, decreases T_o_ by 5.13 K and 5.26 K, respectively, for C = 0% and 4%. Furthermore, increasing C reduces T_o_, since higher EPCM fractions increase the effective heat capacity of the emulsion, allowing for more heat from the LIB to be absorbed as latent energy. Specifically, at Re = 50 and 100, an increase in C from 0% to 4% results in a decrease in T_o_ of 1.26 K and 1.39 K, respectively. As shown in Fig. 5, TLIB exhibits a similar trend, although values are lower because both inlet and outlet temperatures are incorporated into the calculation. For example, if Re is increased from 50 to 100, TLIB is reduced by 2.7 K (C = 0%) and 2.48 K (C = 4%). Similarly, increasing C from 0% to 4% decreases TLIB by 1.1 K and 0.88 K, respectively, at Re = 50 and 100.

**Fig. 6:**
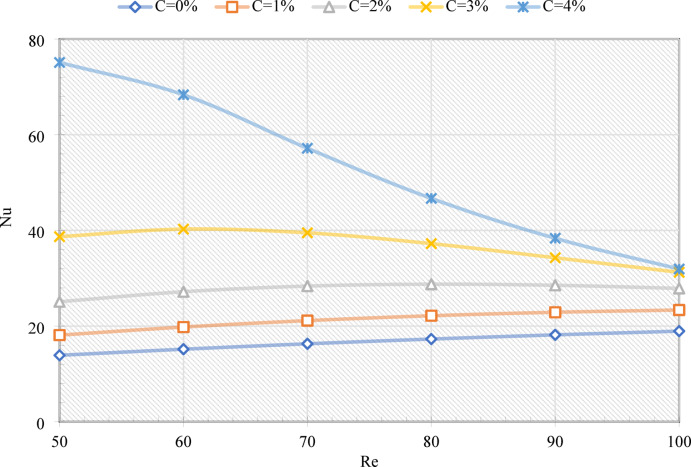
The Nu on the wall of the MHT for various Re and C.

Figure 6 illustrates the variation in Nu with Re and C. For pure water (C = 0%), Nu rises steadily with Re, due to enhanced convective heat transfer; more specifically, Nu increases by 36.35% as Re increases from 50 to 100. At C = 1%, the trend is similar to that of pure water, but with consistently higher values. As an example, increasing C from 0% to 1% enhances Nu by 30.3% at Re = 50 and 23.3% at Re = 100, which indicates that stronger effects are observed at lower Re values. After a C = 2% increase in Re, Nu increases until ~ 70 before stabilizing, as EPCMs begin to exit the MHT without fully melting. However, there are notable improvements observed: Nu increases by 80.6% at Re = 50 and 47.1% at Re = 100 in comparison with pure water. At C = 3%, Nu initially increases at low Re but then decreases as Re increases, decreasing by 21.3% between Re = 50 and 100. Nevertheless, EPCMs contribute significantly to Nu, with a 178.4% boost at Re = 50 and a 68.7% boost at Re = 100. The Nu fraction exhibits a marked decline with C = 4%, since the high EPCM fraction lowers the emulsion temperature and limits the melting of PCM. As an example, Nu increases 3.81-fold at Re = 50 and 0.68-fold at Re = 100 when compared to water, but the increase in Re from 50 to 100 is accompanied by a significant reduction of 57.2%. These results indicate that, despite moderate C values having a positive effect on heat transfer, excessive loading and high Re result in reduced effectiveness due to incomplete melting, highlighting the competing effects of convection and PCM phase-change.

**Fig. 7:**
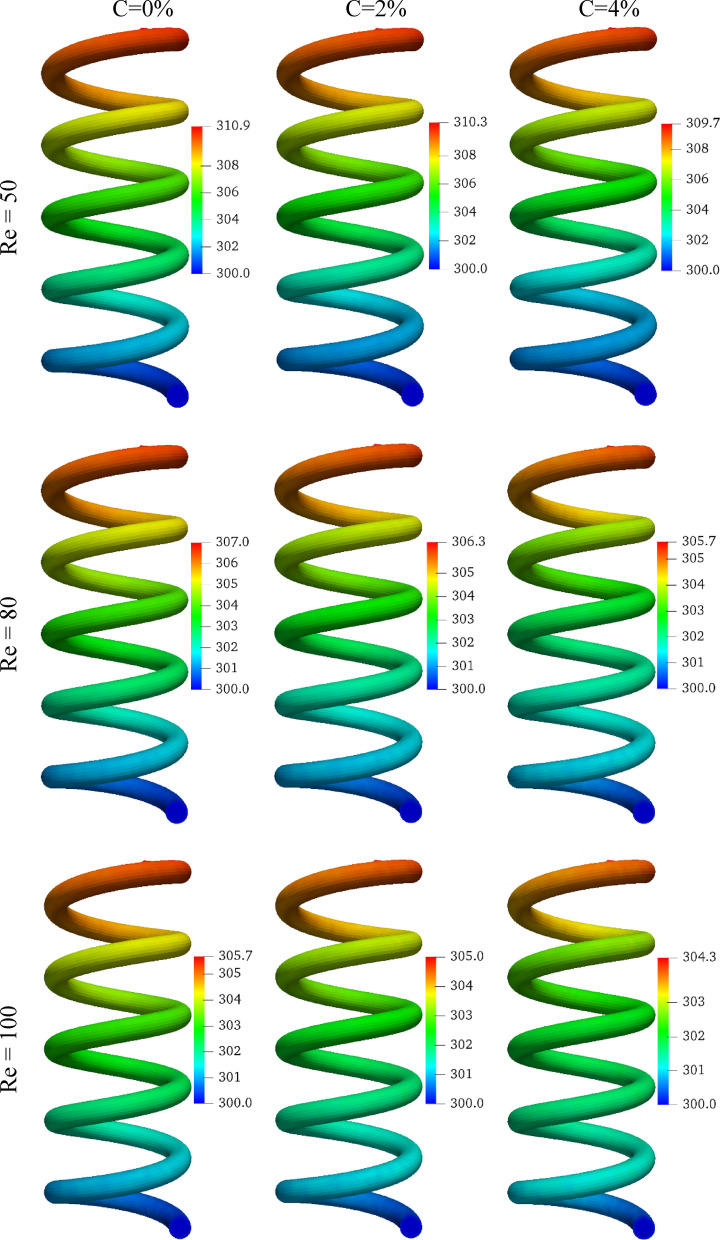
The temperature distribution on the wall of the MHT for various Re and C values.

Figure 7 illustrates the temperature distribution along the wall of the MHT based on different values of Re and C. The temperature of the wall increases gradually from the inlet (300 K) to the outlet of the MHT, where the coolant exits. Increasing Re consistently reduces wall temperatures for all C, since higher flow rates enhance convective heat transfer and promote efficient heat dissipation. As an example, raising Re from 50 to 100 decreases the maximum wall temperature by 5.2 K, 5.3 K, and 5.4 K when C = 0%, 2%, and 4%, respectively. In addition to lowering wall temperatures, increasing C also facilitates the absorption of heat by the EPCMs in their latent state, resulting in a reduction in the thermal load carried as sensible heat. Adding 4% EPCM, for example, decreases the maximum wall temperature by 1.2 K, 1.3 K, and 1.4 K when Re = 50, 80, and 100. The reductions in heat absorption capacity confirm that higher EPCM loading improves heat absorption capacity, although the magnitude of improvement decreases as Re increases, because PCMs have less time to melt completely.

**Fig. 8:**
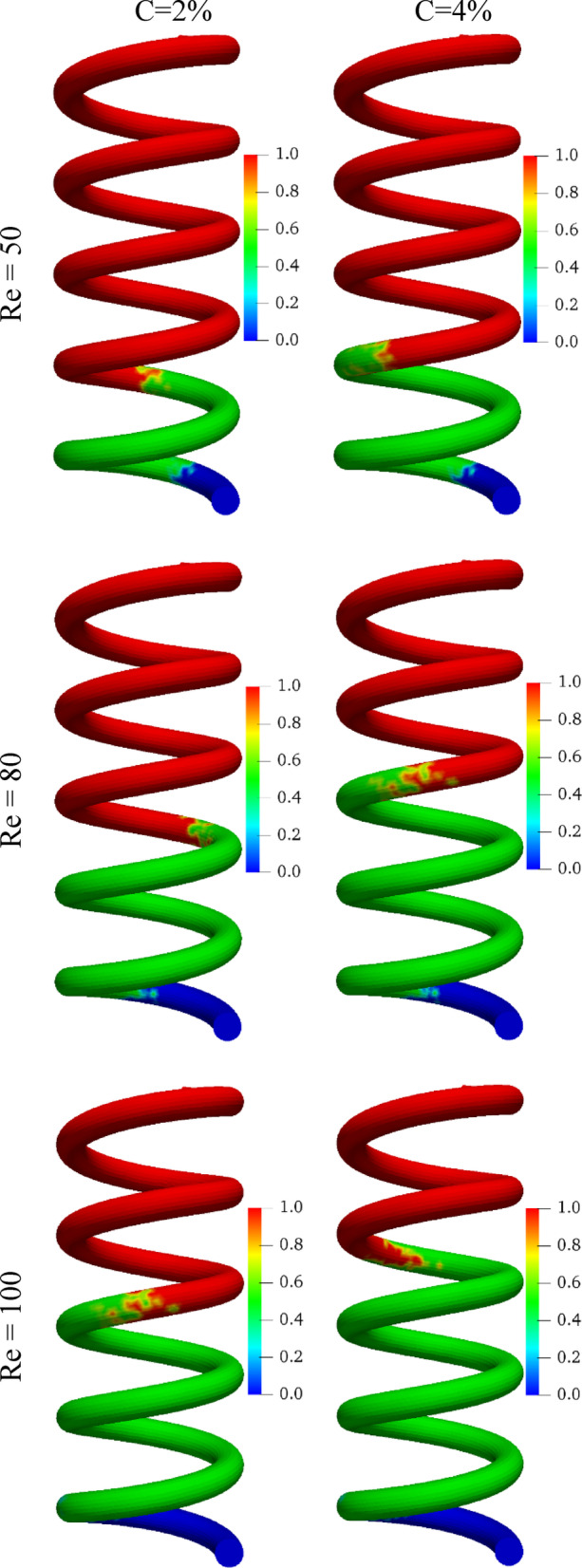
The molten EPCM distribution inside the MHT for various Re and C.

Figure 8 illustrates how EPCM core melting is distributed along the MHT at different Re values between 2 and 4%. The results of the study indicate that increasing Re prolongs the melting transition length (highlighted in green). It is thought that this behavior arises from the fact that higher flow rates enhance the removal of convective heat, thereby lowering the bulk emulsion temperature. Thus, not all EPCM particles reach their melting temperature before exiting the MHT. For instance, increasing Re from 50 to 100 results in an approximately 2.5 pitch lengthening of the transition region for both C = 2% and C = 4%. A similar effect is observed when C is increased. A higher C value increases the emulsion’s effective heat capacity, further suppressing the local temperature rise. In consequence, the melting of EPCM cores is delayed, and thus, the transition region becomes longer. For example, increasing C from 2 to 4% extends the transition length by approximately 0.5 pitches across all examined Re values. Accordingly, these findings highlight the trade-off between improving thermal capacity with additional EPCM loading and the risk of incomplete melting, which may reduce the efficiency of latent heat utilization.

In order to fully assess the thermal-hydrodynamic performance of the proposed EPCM-water emulsion cooling system, a performance index (PI) is calculated as follows:

13 $$PI = ~\frac{{\left( {{{Nu} \mathord{\left/ {\vphantom {{Nu} {Nu_{0} }}} \right. \kern-\nulldelimiterspace} {Nu_{0} }}} \right)}}{{\left( {{{fr} \mathord{\left/ {\vphantom {{fr} {fr_{0} }}} \right. \kern-\nulldelimiterspace} {fr_{0} }}} \right)^{{1/3}} }}$$

**Fig. 9:**
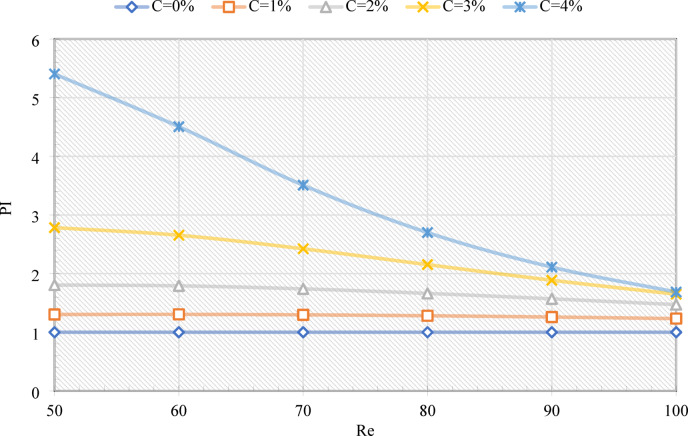
The PI plot of the proposed system for various C and Re values.

In this context, Nu₀ and fr_0_ represent the Nusselt number and friction factor of pure water at equivalent Reynolds numbers, respectively. Figure 9 illustrates the relationship between the Performance Index (PI) and both C and Re. As anticipated, the control case of pure water (C = 0) yields a PI value approximating unity, indicating no significant enhancement. The introduction of EPCM elevates the PI above 1, confirming that the enhancement in heat transfer efficacy surpasses the concomitant increase in frictional losses. The influence of concentration is more pronounced at lower Reynolds numbers, where the extended residence time of the coolant allows for a larger proportion of EPCM particles to undergo a complete phase transition. For instance, the addition of 4% EPCM increases the PI from 1.0 to 5.41 at Re = 50, whereas an identical concentration only elevates the PI from 1.0 to 1.68 at Re = 100. This trend reflects the diminished melting time available at high flow rates, which constrains latent heat contribution. The effect of the Reynolds number aligns with the behavior of the Nusselt number: generally, increasing Re decreases PI, as the relative improvement in heat transfer becomes less dominant compared to the increase in frictional resistance. For example, elevating Re from 50 to 100 reduces PI from 1.31 to 1.23 at C = 1%, and from 5.41 to 1.68 at C = 4%. These findings underscore that optimal performance is achieved at moderate Reynolds numbers with moderate EPCM loading, where sufficient melting occurs without incurring excessive pressure penalties. This balance represents the ideal operating conditions for maximizing heat transfer efficiency while minimizing frictional losses in the system.

### Practicality and utilization

In terms of practical application, the proposed EPCM–water helical tube design offers significant potential for integration into cylindrical cell modules. The helical tubes are readily manufactured using established techniques such as micro-extrusion or additive manufacturing. They are then wrapped around cells to maximize the surface area of contact. Emulsions containing EPCM at low concentrations (≤ 4%) are commercially viable and provide stable operation with manageable pumping power requirements. In addition, the design is compatible with existing coolant circulation systems, making it suitable for both single-cell and pack-level BTMS. However, while further experimental validation and long-term stability studies are needed, the results suggest that the proposed design can maintain battery temperatures within the safe window (20–40 °C) under realistic operating conditions, thereby enhancing system performance and safety.

## Conclusions

This study addresses the challenge of efficient BTMS through the innovative use of EPCM dispersed in water and circulated within an MHT directly surrounding the LIB. Contrary to previous works, which investigated EPCM suspensions in generic mini-channels, the present configuration maximizes the surface contact with the cylindrical cell, thereby optimizing the utilization of sensible and latent heat. This study has demonstrated significant improvements in thermal performance. Changing Re from 50 to 100 reduces T_o_ by 5.26 K at 4%, while changing C from 0 to 4% at Re = 100 decreases To by 1.39 K. Nu exhibits significant enhancement, with a 36.35% increase when Re increases from 50 to 100 and a 178.4% increase when C increases from 0 to 4% at Re = 50. Furthermore, the transition length of EPCM melting increases 2.5 pitches as Re increases from 50 to 100, and by 0.5 pitches as C increases from 2 to 4%, indicating a strong effect of both flow rate and particle loading on the dynamics of latent heat absorption. According to these findings, while higher C values increase the effective heat capacity of the emulsion, excessive loading can delay the completion of full melting and reduce the efficiency of latent heat utilization. From a system-level perspective, the EPCM–water MHT cooling design effectively maintains TLIB within the recommended safe operating window (20–40 °C), thereby minimizing the risk of thermal runaway and extending cell life. Aside from single-cell applications, this strategy may also be extended to LIB modules or packs, where compact integration and scalability are crucial. Prospects include extending the investigation to higher C values and Re values, where there will be a trade-off between viscosity, pumping power, and incomplete melting. Additionally, coupling this approach with hybrid BTMS (such as air–liquid or PCM–liquid combinations) may yield further efficiency gains and practical implementation pathways for electric vehicles and high-power energy storage. Finally, this study highlights the novelty and practicality of using EPCM emulsions for LIB cooling channels, providing a promising avenue toward a safer, more efficient, and longer-lasting LIB.

## Data Availability

The datasets used and/or analyzed during the current study are available from the corresponding author upon reasonable request.

## Abbreviations

BTMS: Battery thermal management system
CFD: Computational fluid dynamics
EPCM: Encapsulated phase change material
FVM: Finite volume method
LIB: Lithium-ion battery
MHT: Mini helical tube
PCM: Phase change material
PDE: Partial differential equation
PI: Performance index
PMMA: Polymethyl methacrylate
PTMS: Passive thermal management system
SIMPLE: Semi-implicit method for pressure-linked equations
TMS: Thermal management system
TPP: Thermophysical property

## List of symbols

*C*: Concentration (–)
*C*_*p*_: Specific heat capacity (J/kg K)
$\:\dot{g}$: Volumetric heat generation (W/m^3^)
H: Height (m)
*h*: Latent heat (kJ/kg)
*k*: Thermal conductivity (W/m K)
*L*: Cavity size (m)
*Nu*: Nusselt number (–)
*P*: Pitch (m)
*p*: Pressure (N/m^2^)
*r*: Radius (m)
*Re*: Reynolds number (–)
*T*: Temperature (K)
*T*_*mr*_: Melting temperature range (K)
*T*_*m*_: Melting temperature (K)
***V***: Velocity vector (m/s)
*x* and *y*: Cartesian coordinates (m)

## Greek symbols

*λ*: Melting fraction (–)
*µ*: Dynamic viscosity (kg/m s)
*ρ*: Density (kg/m^3^)
*ω*: Core-shell weight ratio (–)

## Subscripts

*c*: Core
*LIB*: LIB
*ref*: Reference
*e*: Emulsion
*i*: Inlet
*o*: Outlet
*s*: Shell
*bf*: Base fluid
